# *In vitro* modeling of dendritic atrophy in Rett syndrome: determinants for phenotypic drug screening in neurodevelopmental disorders

**DOI:** 10.1038/s41598-020-59268-w

**Published:** 2020-02-12

**Authors:** Elisa Nerli, Ottavia Maria Roggero, Gabriele Baj, Enrico Tongiorgi

**Affiliations:** BRAIN Center for Neuroscience, Department of Life Sciences, University of Trieste, 34127 Trieste, Italy

**Keywords:** Cellular neuroscience, Autism spectrum disorders

## Abstract

Dendritic atrophy, defined as the reduction in complexity of the neuronal arborization, is a hallmark of several neurodevelopmental disorders, including Rett Syndrome (RTT). RTT, affecting 1:10,000 girls worldwide, is mainly caused by mutations in the *MECP2* gene and has no cure. We describe here an *in vitro* model of dendritic atrophy in Mecp2^−/y^ mouse hippocampal primary cultures, suitable for phenotypic drug-screening. Using High-Content Imaging techniques, we systematically investigated the impact of culturing determinants on several parameters such as neuronal survival, total dendritic length, dendritic endpoints, soma size, cell clusterization, spontaneous activity. Determinants included cell-seeding density, glass or polystyrene substrates, coating with poly-Ornithine with/without Matrigel and miniaturization from 24 to 96-half surface multiwell plates. We show that in all plate-sizes at densities below 320 cells/mm^2^, morphological parameters remained constant while spontaneous network activity decreased according to the cell-density. *Mecp2*^−/y^ neurons cultured at 160 cells/mm^2^ density in 96 multiwell plates, displayed significant dendritic atrophy and showed a marked increase in dendritic length following treatment with Brain-derived neurotrophic factor (BDNF) or Mirtazapine. In conclusion, we have established a phenotypic assay suitable for fast screening of hundreds of compounds, which may be extended to other neurodevelopmental diseases with dendritic atrophy.

## Introduction

Dendritic atrophy, defined as the reduction in branching complexity of neuronal dendritic processes, is a typical hallmark of several neurodevelopmental disorders^1–4^. With an incidence of 1:10,000 newborn girls, Rett syndrome (RTT) is one of the most common neurodevelopmental diseases with mental retardation in females^5^. In 90–95% of cases, the disease is caused by sporadic mutations in the *MECP2* gene^6^. RTT is characterized by a period of early normal development followed by a regression phase, leading to loss of speech and acquired motor skills, presence of stereotypical hand movements, seizures and microcephaly^6^. Presently, there is no cure for Rett syndrome.

Due to microcephaly, the brains of RTT patients show more closely packed neurons^7^ and reduced dendritic complexity has been described in cerebral cortex, hypothalamus and hippocampus^8–10^. The dendritic atrophy observed in the cortex of RTT patients has been related to dysfunctions of neural networks and intellectual disability^9^, similarly to other neurodevelopmental disorders such as Fragile-X syndrome and Down syndrome^1,7^. Accordingly, modelling dendritic atrophy for these diseases is extremely important.

Notably, in mouse models for Rett syndrome, reduced brain dimensions and dendritic atrophy has been found in the same brain regions as in humans^11,12^. We previously showed that *in vivo* treatment of *Mecp*2^−/y^ mice with the antidepressant Mirtazapine was able to rescue cortical thickness, neuronal soma area and apical dendrites diameter counteracting breathing abnormalities and restoring normal anxiety behavior^13^. However, Mirtazapine was unable to fully rescue the phenotype observed in this animal model of RTT, thus prompting the question if there would be other drugs, already approved for human usage, that could be repurposed for treating this syndrome. We previously investigated the development of wild type (WT) and *Mecp2*^−/y^ hippocampal neurons in cultures in 24 multiwell (MW) plates from days *in vitro* (DIV) 1 to DIV 15, demonstrating that RTT neurons showed a deficit in neuronal development between DIV 6–15. In particular, *Mecp2*^−/y^ hippocampal neurons displayed reduced total dendritic length (TDL) and lower complexity of secondary or higher order dendrites, peaking at DIV 12^14^. However, neuronal cultures grown in 24 MW plates are unsuitable as drug screening assay to test a large library of compounds.

In this study, we aimed to develop an *in vitro* model of dendritic atrophy in *Mecp2*^−/y^ mouse hippocampal primary cultures, suitable as a phenotypic drug screening assay. To reach this goal, we used High Content Imaging techniques for automating the process and we systematically investigated how different culture conditions affect morphological and functional parameters within the cultures.

## Results

In order to establish a phenotypic drug screening assay specific for the Rett syndrome, we exploited an *in vitro* model previously established in our laboratory^14^. This model was originally developed using hippocampal neurons seeded at 640 cells/mm^2^ on 13 mm diameter glass coverslips, coated with poly-L-Ornithine and Matrigel^14^. In these conditions, the hippocampi explanted bilaterally from one P0–P1 mouse were sufficient to generate cultures in 6 wells of a 24 Multiwell (MW) plate. In order to obtain culture conditions suitable for a drug screening, we evaluated the possibility to miniaturize the cultures to both reduce the number of animals needed and increase the number of compounds that could be tested using a single animal. Accordingly, we carried out a systematic analysis of culture conditions to define the most appropriate method. To describe neuronal morphology, we evaluated three main parameters, 1) the Average Total Dendritic length (TDL) given by the sum of the length of the entire dendritic arborization of a neuron; 2) the Number of Endpoints consisting in the number of terminal points counted at the end of visible dendrites labeled by anti-MAP2 immunofluorescence. The latter parameter, in particular, represents an index of dendritic arborization complexity and recapitulates the number of terminal branchings of a neuron. Finally, 3) the Soma Area, which is the average of the area of the soma of each neuron expressed in *µ*m^2^.

### Effect of substrate materials and cell-seeding density

**Figure 1:**
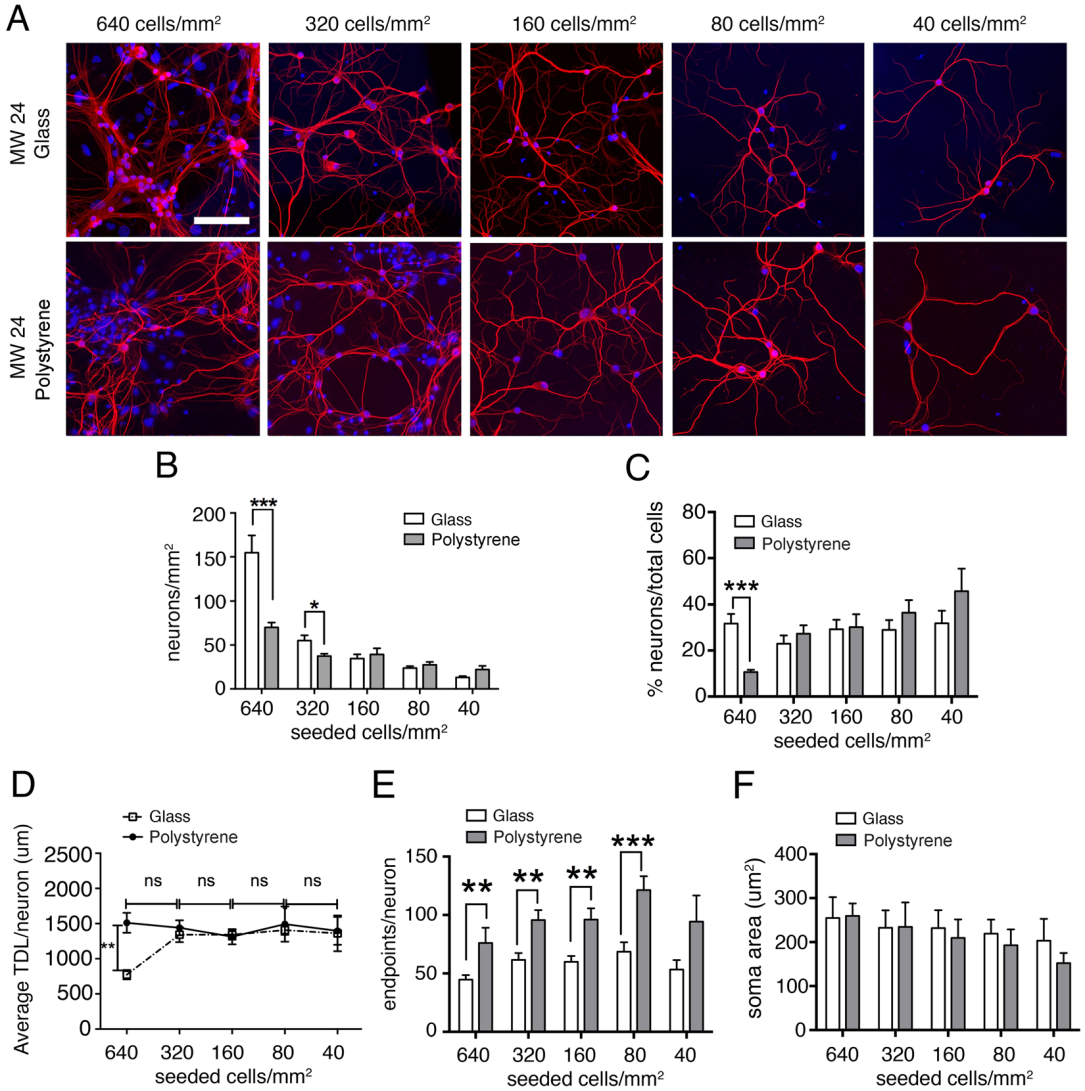
Effect of glass or polystyrene substrates and cell-seeding density on dendrites of wild-type mouse hippocampal neurons. (**A**) DIV 12 mouse hippocampal neurons immunostained for dendrites cytoskeleton (MAP2, red) and nuclei (Hoechst, blue) plated at different seeding cell densities on glass coverslips (top lane) or polystyrene plates (bottom lane) (Scale bar = 100 *µ*m). (**B**) Number of neurons per mm^2^ counted at DIV 12 for the different seeding cell densities. Data are expressed as mean ± SEM, n = 4 cultures per condition on glass, n = 3 cultures per condition on polystyrene. (**C**) Percentage of neurons (%) normalized on the number of counted viable nuclei (total cells), (**D**) average Total Dendritic Length (TDL) per neuron (*µ*m), (**E**) average number of dendrites endpoints per neuron and (**F**) average neuronal soma area (*µ*m^2^). Number of neurons measured ranged from 1000 for the highest seeded cell density, to 200 neurons for the lowest seeded cell density. Unpaired t-test to compare the different substrates at same cell density, *P ≤ 0.05, **P ≤ 0.01, ***P ≤ 0.001. One-way ANOVA was used to compare TDL, endpoints and soma area at different dilutions (**D,E**), ns = not significant difference.

Since previous studies found that dendritic development is affected by culture conditions^15–18^ we investigated the effect of different culture substrates and cell density on neuronal morphology. Cultures were analyzed at DIV 12, corresponding to the time point at which *Mecp2*^*−/y*^ (MeCP2-KO) neurons display the highest morphological deficit with respect to wild type (WT) neurons^14^. Experiments in this first part of the study were carried out with WT mice only, to reduce the number of suffering animals used in this study. To investigate the influence of the substrate on neuronal morphology, hippocampal cells were plated at the cellular density of 640, 320, 160, 80 or 40 cells/mm^2^ in 24-well plates either on glass coverslips as in Baj *et al*. 2014 (Fig. 1A, top row) or directly on polystyrene (Fig. 1A, bottom row). At DIV 12, we performed a morphological analysis aimed at quantifying the number of neurons/mm^2^, the percentage of neurons over total cells, the average TDL, the number of endpoints and the soma area per neuron. As expected, reducing the number of seeded cells led to a reduction in the number of neurons on both substrates (Fig. 1B). Nevertheless, the percentage of neurons over total cells (Fig. 1C) remained constant at cell densities below 320 cells/mm^2^. In high density-cultures (640 cells/mm^2^), the number of neurons over total cells on glass was significantly higher than on polystyrene. The average TDL per neuron, represented in Fig. 1D, resulted to be a very constant parameter at all cell densities on polystyrene, although at 640 cells/mm^2^, neurons on glass coverslips presented a significantly shorter TDL than on polystyrene. The number of endpoints was constant at all cell densities for each substrate (Fig. 1E), but was significantly higher for neurons plated on polystyrene. The soma area was constant (200–300 *µ*m^2^) across the different cell densities and substrates (Fig. 1F). In conclusion, at 640 cells/mm^2^ we found higher cell and neuron survival, but lower TDL on glass with respect to polystyrene, while at cell densities below 320 cells/mm^2^ the percentage of neurons/total cells, TDL, endpoints and soma size remained substantially unchanged, with a slightly higher variability at 40 cells/mm2, which led us to exclude this cell density from further experiments. Of note, changing the seeding substrate in 24 MW plates from glass to polystyrene, led to higher values of endpoints (Fig. 1E). This finding was likely caused by an artifact due to the thick plastic 24 MW plates which presented a much higher “background intensity” that was not found in other types of multiwell plates, as further discussed in the subsequent section “Effect of different well size”. In addition, we show that it is possible to use a polystyrene substrate without glass coverslip.

### Effect of different coating protocols

**Figure 2:**
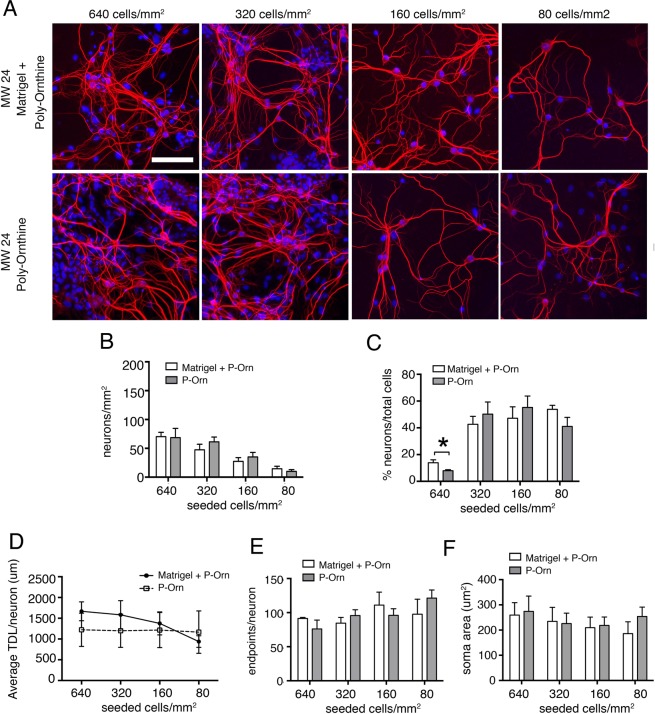
Effect of different coating substrates on dendrites of wild-type mouse hippocampal neurons. (**A**) DIV 12 mouse hippocampal neurons immunostained for dendrites cytoskeleton (MAP2 red) and nuclei (Hoechst blue) at different seeding cell densities with different coatings, such as Matrigel and poly-L-Ornithine (top lane) or only poly-L-Ornithine (bottom lane) (Scale bar = 100 *µ*m). (**B**) Quantitative data on the number of neurons per mm2 counted at the different seeding cell densities. Data are expressed as mean ± SEM, n = 3 cultures for each condition. (**C**) Number of neurons (%) normalized on the number of counted viable nuclei (total cells), (**D**) average TDL per neuron (*µ*m), (**E**) average number of dendrites endpoints per neuron and (**F**) average neuronal soma area (*µ*m^2^). Number of neurons measured ranged from 1000 for the highest concentration seeded to 200 neurons for the lowest concentration seeded. Unpaired t-test to compare the different coating substrates at same concentration, *P ≤ 0.05; One-way ANOVA to compare TDL, endpoints and soma area at different dilutions (**D–F**).

Matrigel, in combination with poly-Ornithine or poly-Lysine, is usually employed as substrate for cell adhesion. In the next experiments, we evaluated the possibility to simplify the coating protocol, in particular on removing Matrigel, a substance that mimics the extracellular compartment, made of proteins extracted from the Engelbreth-Holm-Swarm (EHS) mouse sarcoma. Cells were seeded on polystyrene 24-well plates at different cell densities and wells were coated with Matrigel and poly-L-Ornithine (Fig. 2A, top row) or with poly-L-Ornithine, only (Fig. 2A, bottom row). We observed no differences in the number of neurons/mm^2^ on the two different coatings at each cell-seeding density, nor in the ratio of neurons over total viable cells. However, at the density of 640 cells/mm^2^ the number of neurons over total cells was smaller than at the other cell densities, most likely due to an overgrowth of glial cells. We also found no changes in morphological parameters, therefore we concluded that the elimination of Matrigel from the coating protocol does not affect neuronal morphology and attachment to the substrate.

### Effect of different well size

**Figure 3:**
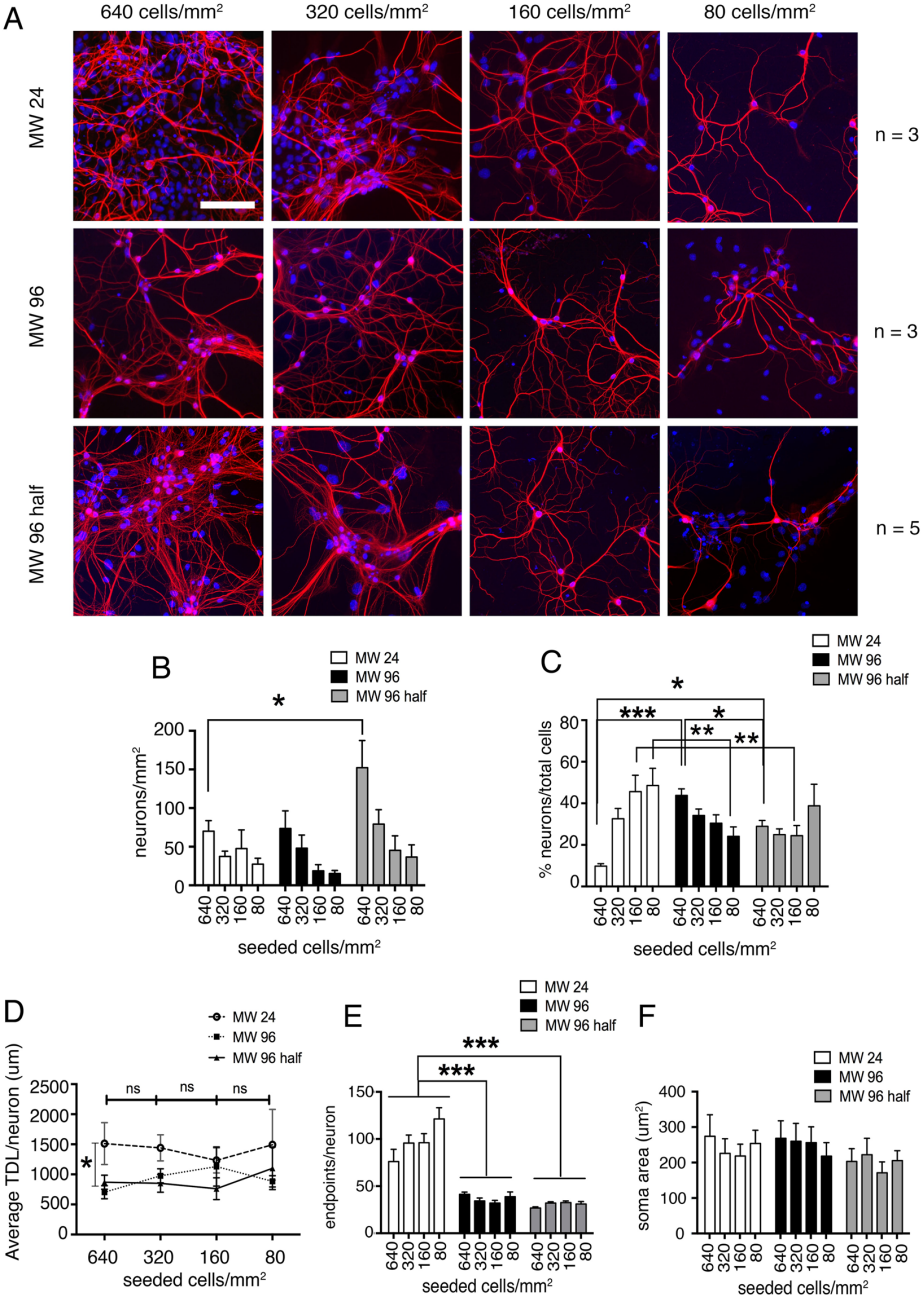
Effect of different well-size on dendrites of wild-type mouse hippocampal neuron. (**A**) DIV 12 mouse hippocampal neurons immunostained for dendrite cytoskeleton (MAP2 red) and nuclei (Hoechst blue) plated at different seeding cell densities in different polystyrene plates: MW 24 (top line), MW 96 (middle line) and MW 96 half-surface (bottom line). (Scale bar = 100 *µ*m) (**B**) Quantitative data on the number of neurons per mm^2^ counted at the different seeding cell densities. Data are expressed as mean ± SEM, n = 3 cultures per condition on MW 24, n = 3 cultures per condition on MW 96 and n = 5 cultures per condition on MW 96 half. (**C**) Number of neurons (%) normalized on the number of counted viable nuclei (total cells), (**D**) Average TDL per neuron (*µ*m), (**E**) average number of dendrite endpoints per neuron and (**F**) average neuronal soma area (*µ*m^2^). Kruskal-Wallis test corrected for multiple comparisons (Dunn’s correction) on panel C. Two-way ANOVA corrected for multiple comparisons (Dunn’s correction) to compare the different plates and cellular densities (**B–F**). For both, P > 0.05, *P ≤ 0.05, **P ≤ 0.01, ***P ≤ 0.001.

We then examined the neuronal morphology following miniaturization of the cultures from 24-well plates to 96 and 96 half-surface well plates (Fig. 3A). We seeded cells on poly-L-Ornithine at the previously used cell densities and we investigated the same morphological parameters. The number of neurons/mm^2^ was comparable among the same cell densities in the different types of plates, with the exception of the 640 cells/mm^2^ at which there was a significant difference between 24-well plates and 96 half-surface well plates with higher number of neurons/mm^2^ in 96 half-surface well-plates (Fig. 3B). The analysis of the percentage of neurons/total number of cells provided a more complex picture (Fig. 3C). On 24 MW polystyrene plates, at 640 cells/mm^2^ the percentage of neurons was significantly smaller than at other cell densities and increased progressively at lower cell densities. In contrast, on 96 MW plates, at 640 cells/mm^2^ the percentage of neurons was higher and decreased progressively at lower cell densities, while on 96-half-surface plates there was no significant difference among the different cell densities (Fig. 3C). The average TDL measured presented a comparable trend between 24, 96 and 96-half surface well plates, in particular at the density of 160 cells/mm^2^, at which all plate types showed a very similar mean TDL (Fig. 3D). The only significant difference was observed at 640 cells/mm^2^, at which the TDL measured in 24 well plates was significantly higher than in 96 and 96-half surface well plates.

The number of endpoints, as described in Fig. 1, was not influenced by the different cell densities within each types of plates (Fig. 3 panel E). However, when comparing TDL values between the different plate types, we found that 96 MW and 96 MW half-surface-well plates showed a very similar TDL, with values consistent with the original model established on glass coverslips in 24 well plates (compare with Fig. 1). On the contrary, neurons plated on polystyrene 24 well plates had significantly higher TDL with respect to 96 and 96 half-surface well plates, at all cell densities. Thus, the endpoints measure appears to be not reliable on 24 well plates, resulting in a significant difference between this type of plates and the 96 and 96 half-surface plates. This finding suggests that the endpoints measure on 24 polystyrene plates could be an artifact that is absent in 96 and 96 half-surface plates. Soma area was not affected by the different well size and the different densities of seeded cells (Fig. 3F). Although we found a higher variability of the TDL measurements in 24 well plates with respect to 96 and 96 half-surface plates, we found that the three types of plates induced a similar neuronal growth. In conclusion, miniaturization of the culture does not affect neuronal morphology and the number of neurons present in the cultures plated at cell densities below 640 cells/mm^2^.

### Glial contribution to neuronal morphology

Once chosen the most suitable plate format for a drug screening (i.e. 96 MW), we evaluated the contribution of glial cells to neuronal morphology. In particular, we investigated if the removal of glial cells by Cytosine β-D-arabinofuranoside (Ara-C), an inhibitor of cell replication, could affect neuronal morphology. Hippocampal cells were seeded on 96 well plates on poly-L-Ornithine at the different cell densities previously used and neurons were maintained in culture in presence or absence of 2.5 *µ*M Ara-C (Supplementary Fig. 2A, top and bottom rows respectively). As shown in Supplementary Fig. 2B we observed a higher number of neurons at 640 cells/mm^2^ when cells were grown without Ara-C, while this difference disappeared at lower cell densities. As expected, the percentage of neurons over total alive cells was different between the two conditions (Supplementary Fig. 2C), indicating a higher proliferation of glial cells in cultures that did not receive Ara-C, but this difference was not significant at cellular densities below 160 cells/mm^2^. Interestingly, a different number of neurons over glial cells did not affect the average TDL at the different cellular concentration (Supplementary Fig. 2D), indicating that this is a very robust parameter. Moreover, the number of endpoints and the soma area per neuron remained constant in the different conditions (Supplementary Fig. 2E,F), indicating that the removal of Ara-C did not affect neuronal morphology. Nevertheless, we decided to include Ara-C in our assay in order to be consistent with previous studies in the literature.

### Cellular distribution within wells of different size

**Figure 4:**
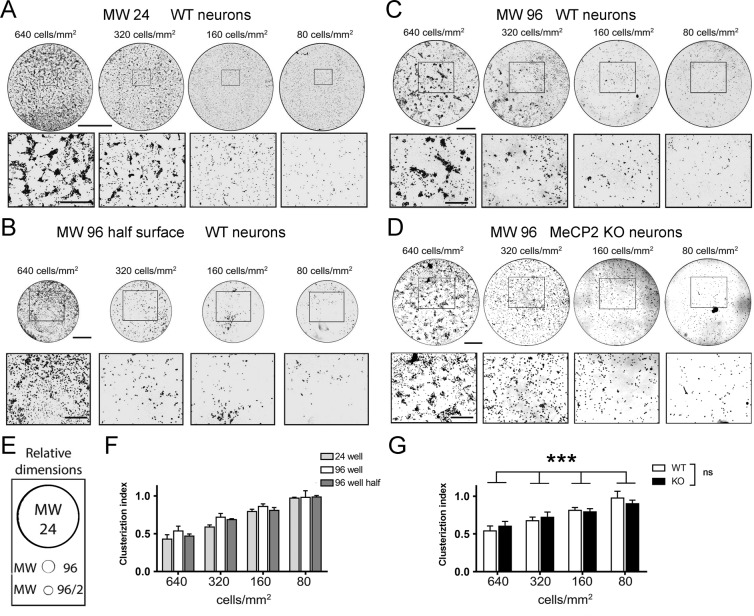
Distribution of seeded cells within wells in 24 MW, 96 MW and 96MW-half surface plates. (**A–D**) Qualitative representation of the distribution of nuclei (Hoechst) in the entire well at the different cell densities of WT and *Mecp2*^−/y^ (MeCP2 KO) hippocampal neuronal cultures at DIV 12. The upper panels show representative images of the wells (pictures show 90% of well surface), the bottom lanes show a close-up magnification in each condition. (**A**) Distribution of WT hippocampal neurons in 24 MW polystyrene plates. Scale bar = 5 mm for the well, 1 mm for the close-up. (**B**) Distribution of WT hippocampal neurons in 96 MW polystyrene plates. Scale bar = 1 mm for the well, 500 *µ*m for the close-up. (**C**) Distribution of WT hippocampal neurons in polystyrene 96 MW half-surface plates. Scale bar = 1 mm for the well, 500 *µ*m for the close-up. (**D**) Distribution of *Mecp2*^−/y^ (MeCP2 KO) hippocampal neurons in 96 MW polystyrene plates. Scale bar = 1 mm for the well, 500 *µ*m for the close-up. (**E**) Actual well dimensions. Relative proportions between panel B and C are retained, while panel A dimensions were reduced. (**F**) Cell Cluster Index for WT hippocampal cultures at the different seeding cell densities in the different plates. (**G**) Comparison of the Cell Cluster Index between WT and *Mecp2*^−/y^ (KO) hippocampal cultures at the different seeding cell densities in 96 well plates. The statistical evaluation was performed using two-way ANOVA to compare the variables genotype and cellular density. n = 3 independent experiments for each cell density and multiwell type, ***P ≤ 0.001.

To run a high-content imaging (HCI) analysis, it is necessary that neurons do not form clusters. Indeed, in case of cell cluster formation, dendrites of different neurons could fasciculate and the software would be unable to measure the dendritic arborization correctly. Therefore, we measured the cellular distribution across the well surface in the different culturing conditions using the *Cell Cluster Index*, which is a number between 0 and 1, where 0 means that cells aggregate into a single large cluster of cells and 1 corresponds to absence of clusterization, i.e. cells assume a sparse distribution within the well (described in “Methods”). The insert in Fig. 4E shows the relative dimensions of a 24, 96 and a 96 half-surface well plate, while Fig. 4A (circles) shows thresholded and binarized images of an entire 24 well plate, at the different seeding cell densities, with corresponding enlargements (rectangles). Figure 4B shows a 96 MW half-surface well plate and Fig. 4C a 96 well plate, at the indicated cell densities. For all conditions, the Cell Cluster Index is shown in Fig. 4F. We observed a high level of clusterization at 640 cells/mm^2^ in all plate types while, by diluting the seeded cells, distribution was sparser and the Cell Cluster Index increased comparably. These results provide evidence that the cellular dispersion in the three plate types tested is independent from the plate size but is affected by cell density. Since in the 96 half-surface well plates, we occasionally found wells completely devoid of neurons due to unexplained cell mortality (data not shown), we finally chose 96 well plates as the most suitable format to establish a drug screening assay.

### Cellular distribution of *Mecp2*^*−/y*^ cells within wells of different size

After scaling down the *in vitro* model from 24 well plates to 96 well plates using WT neurons, we evaluated how the new culturing conditions affect the morphology of neurons with *Mecp2*^*−/y*^ genotype. Thus, we seeded WT and *Mecp2*^*−/y*^ hippocampal cells on 96 MW plates at the different cell densities used in the previous experiments (Fig. 4D) and we performed analysis at DIV 12. Using nuclear staining, we first measured the Cell Cluster Index and we observed a significant difference due to the different cell density, but the genotype was irrelevant with respect to the cellular distribution (Fig. 4G). These findings show that it is possible to reduce the cell seeding density in the assay down to 80 cells/mm^2^ without altering the cellular dispersion of WT and *Mecp2*^*−/y*^ hippocampal neurons within the culturing wells.

### Functional calcium imaging in WT and *MeCP2*^−/y^ cells at different seeding densities

**Figure 5:**
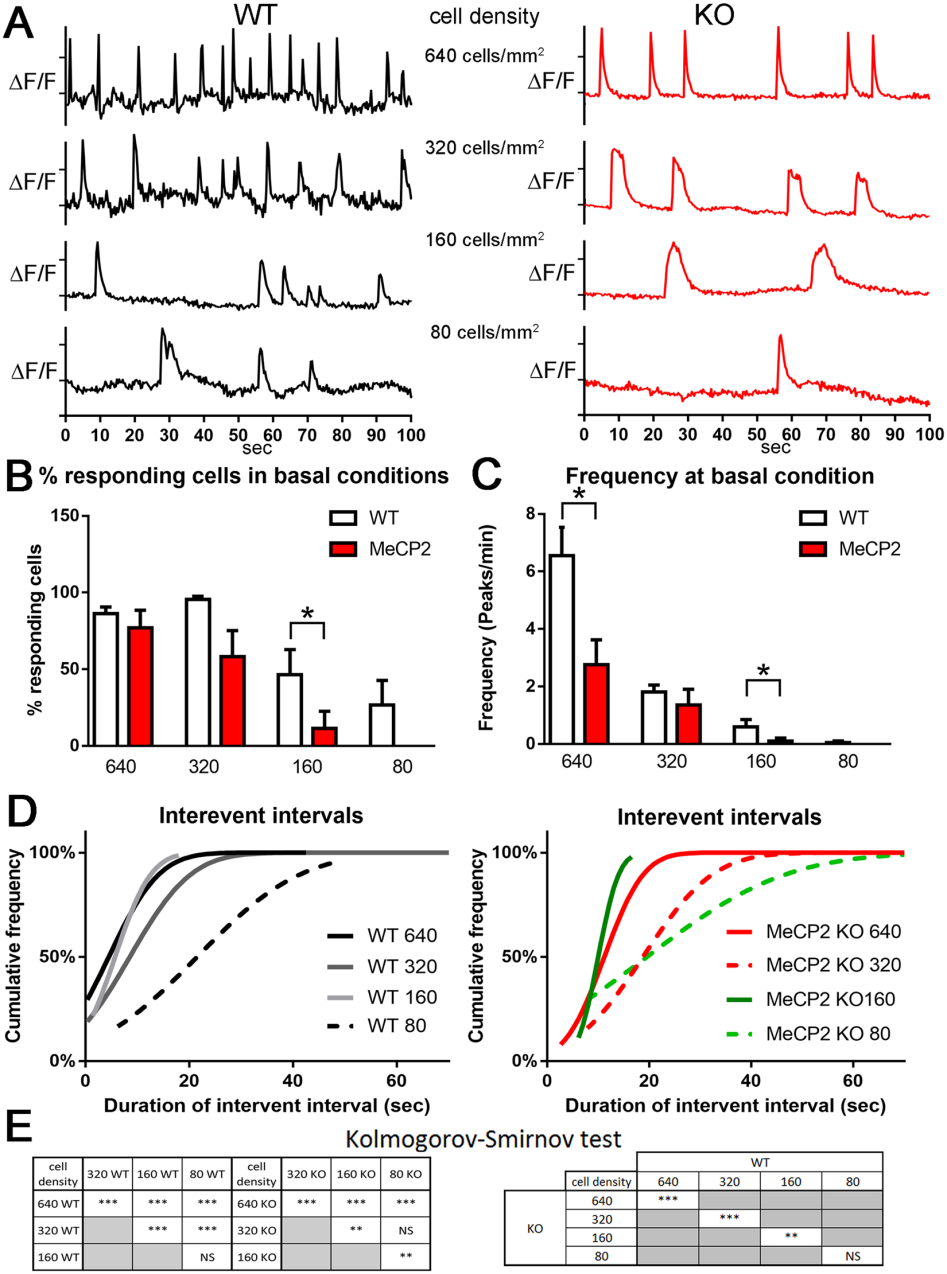
Effect of cell density on network activity measured by Ca^2+^ imaging analysis in WT and *Mecp2*^−/y^ neurons. (**A**) Example of Calcium transients in WT (black traces on the left) and in *Mecp2*^*−/y*^ (KO, red traces on the right) single neurons at each cell density, from the top: 640 cells/mm^2^, 320 cells/mm^2^, 160 cells/mm^2^, 80 cells/mm^2^. Fluorescence values of each pixel was normalized to the background fluorescence (ΔF/F). (**B**) Quantification of the % of responding WT (white columns) and *Mecp2*^*−/y*^ (red columns) hippocampal neurons at basal condition at the different cell seeding densities. (**C**) Representation of number of peak per minute in WT (white columns) and *Mecp2*^*−/y*^ neurons (red columns) at the different cell seeding densities. (**D**) Time during which Ca^2+^ channels remain close between two consequent peaks. Panels show the cumulative frequency of WT (left panel) and *Mecp2*^*−/y*^ (MeCP2 KO, right panel) neurons plated at 640 cells/mm^2^, 320 cells/mm^2^ and 160 cells/mm^2^, 80 cells/mm^2^ (on the right) over the duration of inter-event interval (in seconds). (**E**) Summary of the statistical significance of the data shown in D using Kolmogorov-Smirnov non-parametric test. The left table summarize the differences between different cell densities for the indicated genotype (WO or KO), while the right panel reports the differences between WT and KO (*Mecp2*^*−/y*^) cultures at the same cell density. ***P < 0.001, **P < 0.01, *P < 0.05.

Previous studies have consistently reported a decrease in the frequency of spontaneous Calcium (Ca^2+^) oscillations and in the percentage of cells exhibiting calcium transients in cultured human iPSCs from RTT patients and murine *in vitro* and *in vivo* models^19–21^. To test if the miniaturized cell cultures were able to develop a functional neuronal network at the different seeding densities, we recorded the spontaneous activity with a Ca^2+^ imaging technique^22^. We used a high affinity Ca^2+^ indicator Oregon Green-488 BAPTA, which lead us to detect small Ca^2+^ changes near resting levels^23^. Calcium imaging was performed in WT and *Mecp2*^*−/y*^ cells seeded in 96 well plates at 640, 320, 160 and 80 cells/mm^2^. Spontaneous calcium spike activity was recorded in basal conditions (Fig. 5A) with three different read-outs: the percentage of responding cells out of the total cell population (Fig. 5B); the peak frequency (peak/min) (Fig. 5C) and the inter-event intervals (Fig. 5D), namely the duration of calcium channels closure between two consequent peaks. Interestingly, only WT and *Mecp2*^*−/y*^ neurons seeded at 160 cells/mm^2^ showed a significant difference concerning the percentage of the responding cells in the total cell population (Fig. 5B). Qualitative analysis of the calcium spike frequency (peaks/minute) showed that WT neurons are spontaneously activated with higher frequency in cultures with high densities (640 or 320 cells/mm^2^), while at lower density there was a drastic decrease of the frequency both in WT and *Mecp2*^*−/y*^ neurons (Fig. 5A). However, the quantitative analysis showed that calcium spike frequency is significantly higher in WT than in *Mecp2*^*−/y*^ neurons only at 640 and 160 cells/mm^2^ (Fig. 5C). Third, we measured the inter-event interval (Fig. 5D). The slope of the curves generated for the graphs shown in Fig. 5D are cumulative Gaussian distributions and we used a Boltzmann sigmoidal best-fit (Supplementary Table 1) to extrapolate the slope values from these data. Kolmogorov-Smirnov statistical analysis of the distribution of the inter-event interval curves (Fig. 5E), revealed significant differences between different cell densities in both genotypes (p < 0.001 for all WT combinations, and KO at 640 cells/mm^2^; p < 0.01 for KO 160 versus either 320 or 80 cells/mm^2^), but not between 80 and 160 cells/mm^2^ for WT and 80 and 320 cell/mm^2^ (Fig. 5E, left). Most importantly, significant differences were revealed between WT and KO inter-event interval duration at 640, 320 (p < 0.001), 160 (p < 0.01) cells/mm^2^ but not at 80 cells/mm^2^. Comparison of curves shift, indicated that Ca^2+^ channels tend to remain closed for longer time in *Mecp2*^*−/y*^ with respect to WT neurons at all cell density, suggesting a lower spiking frequency. In fact, the shift of the curve to the left represents a minor closure time of Ca^2+^ channels while a shift to the right represents a longer closure time of Ca^2+^channels. Of note, at 160 cells/mm^2^ curves of both genotypes are shifted to the left with respect to the other cell densities”. In conclusion, these experiments suggest that cell cultures at low densities (80 cells/mm^2^) are not suitable to investigate drug-induced changes because of poor neuronal activity and clearly show that in cultures at 160 cells/mm^2^ it is possible to observe clear differences in network activity between WT and *Mecp2*^*−/y*^ neurons.

### Validation of the assay for detection of neurotrophic effects

**Figure 6:**
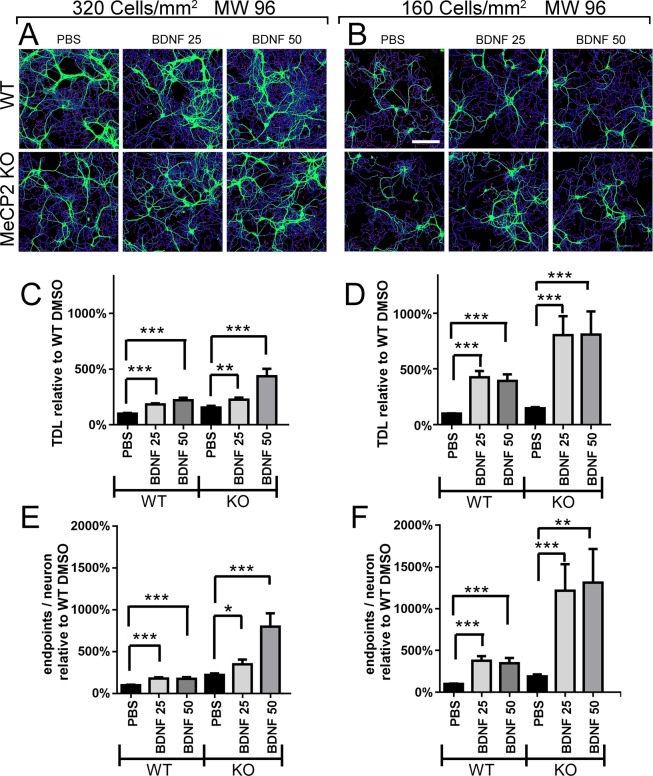
BDNF effects on TDL and endpoints. (**A**) NeuriteQuant morphological analysis of TDL (blue lines) and endpoints (red spots) of WT and MeCP2 KO DIV 12 mouse hippocampal neurons at 320 cells/mm^2^ (n = 3), and (**B**) 160 cells mm^2^ (n = 4) immunostained for dendrite cytoskeleton (MAP2 green). Both WT and MeCP2 KO neurons were treated (from left to right) with PBS, BDNF 25 ng/ml, BDNF 50 ng/ml for 3 days from DIV 9 to DIV 12. (**C,E**) Quantitative data of average TDL per neuron (µm) and average number of endpoints per neuron at 320 cells/mm^2^ and (**D,F**) 160 cells/mm^2^, respectively. N = 3 cultures, each in duplicate wells. Grubbs test for outlier detection and removal with significance level α = 0.05 (two-sided). One-way ANOVA with Dunnett’s multiple comparisons test vs PBS conditions. ***P < 0.001, **P < 0.01, *P < 0.05.

To verify if our assay was able to detect changes in neuronal morphology upon drug treatments, we treated neurons plated in 96 MW plates at the density of 160 cells/mm^2^ with Brain-derived neurotrophic factor (BDNF), which is a well-known enhancer of dendritic arborization even in Rett syndrome^24–28^. BDNF was dissolved in PBS or with the same volume of PBS diluted in the culture medium (Fig. 6). After 3-days treatment, we evaluated the average TDL per neuron at DIV 12 on cells seeded at 320 and 160 cells/mm^2^. Although in control conditions (PBS), we did not detect any deficit in average TDL or endpoints between *Mecp2*^*−/y*^ and WT neurons, we found a marked dose-dependent trophic effect of BDNF 25 ng/ml and 50 ng/ml on both *Mecp2*^*−/y*^ and WT neurons at 320 cells/mm^2^, with a greater average fold change for *Mecp2*^*−/y*^ neurons (Fig. 6C,E). In cultures seeded at 160 cells/mm^2^, the effect of BDNF was even stronger on both genotypes but with identical values for BDNF 25 ng/ml and 50 ng/ml, suggesting a saturating effect (Fig. 6D,F). These results clearly indicates that our method allows to identify a compound exerting a neurotrophic effect.

### Rescue of dendritic atrophy in *Mecp2*^*−/y*^ neurons by Mirtazapine

Drug libraries are typically dissolved in dimethyl sulfoxide (DMSO). The available literature reports a toxic effect in primary neurons exposed to DMSO for 48 hours at concentrations higher than 0.5%^18,29^. Thus, in the next set of experiments we investigated the effects of increasing concentrations of DMSO. As shown in Supplementary Fig. 3, we evaluated the effect of 7 different concentrations of DMSO (from 0.025% to 2%) on WT and *Mecp2*^*−/y*^ neurons, measuring the number of neurons/mm^2^ (Supplementary Fig. 3A) and the average TDL (Supplementary Fig. 3B) with respect to the untreated condition (UNTR). We found significant decrease in average TDL with DMSO at 2% in WT neurons (Supplementary Fig. 3C). In particular, we found no effect DMSO 0.1%, which is the final concentration used for drug screening. Of note, in these conditions, the difference in the average TDL between WT and *Mecp2*^*−/y*^ neurons was significant (p < 0.05) and thus, more evident than in PBS (compare with Fig. 5).

**Figure 7:**
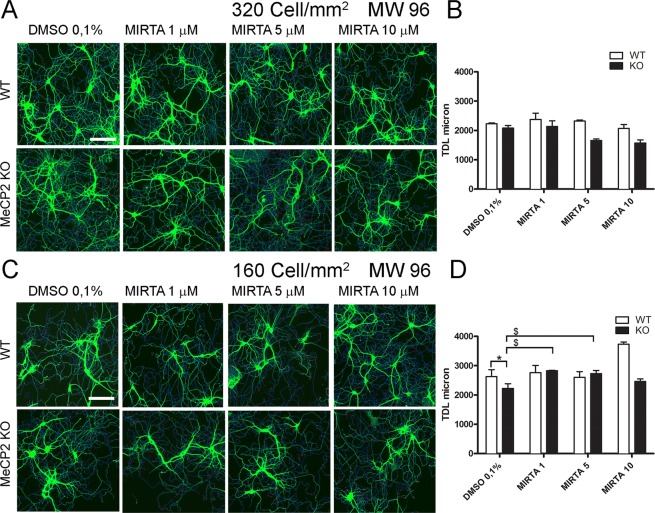
Mirtazapine treatment of *Mecp2*^*−/y*^ hippocampal neurons. DIV 12 mouse hippocampal neurons immunostained for dendrite cytoskeleton (MAP2 green) plated at (**A**) 320 cells/mm^2^, and (**C**) 160 cells mm^2^ (n = 3). (**B**) Quantification of the effect of Mirtazapine on the average TDL of *Mecp2*^*−/y*^ neurons seeded at 320 (n = 3) and (**D**) 160 (n = 3) cells/mm^2^. N = 3 separate experiments, each in duplicate wells. Grubb’s test for outlier detection and removal with significance level α = 0.05 (two-sided). ANOVA and Student’s t-test were used to compare the effect of the drug with vehicle (DMSO 0.1%): P ≤ 0.05 and **P ≤ 0.01.

Then, we validated the assay for a future drug screening using the antidepressant Mirtazapine, which we previously demonstrated to be a positive modulator of dendritic length in *Mecp2*^*−/y*^ mice *in vivo*^13^. We tested this drug in duplicate on neurons seeded at 320 and 160 cells/mm^2^ in 96 MW plates, in three independent experiments. Figure 7 shows the measured changes in average TDL upon treatment with 1, 5, or 10 µM Mirtazapine diluted in 0.1% DMSO. We highlighted a different response of cells seeded at the different cell densities despite a similar average TDL for the control condition (DMSO 0.1%, 2228 ± 58.62 µm at 320 cells/mm^2^ and 2841 ± 7562.6 µm at 160 cells/mm^2^). Specifically, the average TDL of neurons in DMSO 0.1% seeded at 320 cells/mm^2^ was not different between WT and KO neurons and was also not significantly modified by treatment with Mirtazapine (Fig. 7A,B). In contrast at 160 cells/mm^2^, we found a significant reduction in TDL in KO neurons with DMSO 0.1% and a significant rescue effect at all Mirtazapine concentrations tested (Fig. 7C,D). In conclusion, we demonstrated that at a cell density of 160 cells/mm^2^ our method is suitable to highlight a dendritic atrophy in RTT neurons and its rescue following 3 days of treatment with an FDA-approved drug.

## Discussion

In this study, we established a robust, miniaturized *in vitro* model of dendritic atrophy in Rett syndrome suitable as phenotypic drug assay. After having systematically analyzed the impact of culturing conditions on the morphological and functional maturation *in vitro* of primary cultures of wild-type mice hippocampal neurons, we established a culture protocol in 96 well plates format that preserve the normal development and a functional neuronal network at cell densities between 320 and 160 cells/mm^2^. We demonstrate that this assay allows to perform phenotypical drug screening for RTT dendritic atrophy, using High Content Imaging techniques and an open-source software to analyze neuronal morphology.

Previous *in vitro* mouse models reproducing the developmental deficits of the Rett syndrome were developed using high seeding cell-density in the range of 600–800 cells/mm^2^ in 24-well plates^14,27,30–32^. Although a variety of cell-seeding densities ranging from 640 to 40 cells/mm^2^ are currently in use^33–35^, the available literature on phenotypic drug screening suggests that to perform a reliable measure of cellular morphology using automated image analysis tools, the cellular density should be low^18,36^. In neural cultures, low density ensures that neurites do not fasciculate extensively and then can be correctly identified and measured by an analysis software. Ivenshitz and Segal investigated morphological and physiological differences in rat hippocampal primary neurons at high (320 cells/mm^2^), medium (160 cells/mm^2^) and sparse (80 cells/mm^2^) cell densities in 24-well plates. Using Sholl analysis, they reported that neurons in sparse cultures have fewer branching points (12.30 ± 1.61) than in high density cultures (29.80 ± 3.32)^15^. We performed experiments at the same cell-seeding densities and in the same plate format (i.e. 24-well plates) and we found a number of neurons surviving at DIV 12–13 comparable to that of Ivenshitz and Segal^15^, although their initial seeding concentration was 5 times higher than in our experiments. The higher mortality that they observed might be due to the usage of the MEM + B27 culture medium instead of the Neurobasal + B27 as in our study^37,38^. However, in contrast to Ivenshitz and Segal findings, our experiments with WT neurons show that morphological parameters do not change with reduction in cell density. An explanation for this difference could rely in a different culture preparation method, including a different culture medium and contribution of glial cells, as well as a different protocol to perform morphological analysis^15^. Although we found studies that used a very low cellular density for the assay on WT primary neurons^33^, we excluded the seeding cell densities of 80–40 cells/mm^2^ because of their higher variability and poor network activity as demonstrated by our Ca^2+^ imaging experiments.

Of note, in addition to 320, 160, and 80 cells/mm^2^ as in Ivenshitz and Segal (2010)^15^, we also measured neurons from cultures seeded at 640 cells/mm^2^ in 24-well plates as in a previous study^14^. At this cell density, we found that neurons plated on glass appeared to have shorter dendrites than those on 24-well polystyrene plates. We observed that neurons on glass tend to fasciculate easily, leading to an underestimation of average TDL when measuring dendrites using NeuriteQuant. However, by comparing morphological parameters of neurons plated on 24, 96 and 96-half surface polystyrene well plates we show that neuronal morphological parameters are substantially identical for all plate sizes when hippocampal cultures are plated in the range of 320–80 cells/mm^2^. Only for neurons plated on 24-well plates at 640 cells/mm^2^ we found, in addition to low TDL as mentioned above, also a reduced number of endpoints. We argue that this result is likely a software artifact caused by the higher autofluorescence of these plates. Importantly, this artifact is absent in 96-well and 96-halfsurface-well plates although they are also made of polystyrene. Accordingly, we chose 96-well plates for all subsequent investigations.

It was previously demonstrated that WT hippocampal mouse neurons co-cultured with cortical astrocytes lacking Mecp2 display stunted dendrites^30^. In our assay, we found that reduction in the number of astrocytes obtained by incubating the cultures with Ara-C, did not affect TDL and number of endpoints. Our experiments differ from those of Ballas and coworkers (2009)^30^, because they used cortical astrocytes lacking Mecp2, while we used hippocampal astrocytes. A previous study showed that cortical but not hippocampal astrocytes play a neuroprotective role on hippocampal neurons exposed to amyloid β insults^39^. This suggests that astrocytes from different origin may exert a different effect on neuronal development. As Ara-C treatment had no significant effect on neuronal morphology, we decided to include this treatment in the culturing protocol, to be consistent with previous studies^14^.

Dendritic atrophy typically affects the formation of a functional neuronal network thus leading to altered synaptic activity and intellectual disabilities including mental retardation *in vivo*. We evaluated the impact of culturing conditions on neuronal network using morphological and functional analyses, measuring cellular distribution (Cell Cluster Index) and Ca^2+^ imaging, respectively. Using the Cell Cluster Index, we found that in cultures with higher cell densities, especially 640 and 320 cells/mm^2^, neurons tend to aggregate into large cell clusters, while at 160 and 80 cells/mm^2^ neurons are sparser and the majority of them appear isolated. For this reason, we preferred 320 or 160 cells/mm^2^ as seeding concentration for the drug screening assay.

Ca^2+^ imaging is defined as the major complementary approach to investigate multi-neuronal activity^40^. In agreement with a previous study suggesting that cellular concentration affects network activity^15^ we found that Ca^2+^ activity was more intense at higher cell densities, namely 640 and 320 cells/mm^2^, while at a density of 160 cells/mm^2^ was markedly reduced and at 80 cells/mm^2^ was almost completely abolished. Importantly, in *Mecp2*^*−/y*^ neurons, Ca^2+^ activity showed decreased frequency and interevent interval with respect to WT neurons, but this difference was significant only at the density of 160 cells/mm^2^. We could not extrapolate any information about the amplitude and the height of the peaks, because BAPTA does not allow to perform a ratiometric measurement of the calcium wave^15^, therefore we could not quantify the amount of calcium released per peak. A previous study established a connection between Calcium regulation and dendrites retraction in the respiratory nucleus of -*Mecp2* mutant mice^21^. In particular, they reached the conclusion that a small neuronal network (‘small-world’ simulation model) is virtually equal to a larger network in generating stable rhythmic Calcium patterns, causing no effect on dendrites. In contrast, removal of only a few connections gives rise to large spontaneous transients and causes a significant decrease in neuronal network connectivity^21^. Interestingly, in -*Mecp2* KO neurons plated at the density of 160 cells/mm^2^ we observed large transients similar to those observed by Mironov and colleagues which may explain why a significant decrease in TDL between WT and KO was observed at 160 cells/mm^2^ cell density but not at 320 cells/mm^2^ (compare Fig. 7A,B).

As a proof-of-concept drug screening, we tested BDNF and Mirtazapine on *Mecp2*^*−/y*^ neurons in a 96 well plate format. We chose to seed cells at the densities of 320 and 160 cells/mm^2^, under the working hypothesis that a different neuronal network could respond differently to drug treatments. We demonstrate that 160 cells/mm^2^ density cultures incubated with vehicle (0.1% DMSO) display a significant dendritic atrophy with respect to WT and show an evident response to treatment with Mirtazapine. In contrast, medium-density cultures 320 cells/mm^2^ did not show neither a deficit in vehicle conditions nor a response to drug treatment with Mirtazapine. Of note, cultures incubated with PBS instead of DMSO, did not show dendritic atrophy although BDNF diluted at various concentrations in PBS had a marked trophic effect on both WT and *Mecp2*^*−/y*^ neurons. Taken together, these results strongly indicate that phenotypic effects of drugs on *Mecp2*^*−/y*^ neurons are more easily detectable using slightly harsher culturing conditions such as those determined by the 160 cells/mm^2^ seeding density and 0.1% DMSO. Indeed, among all the culturing conditions tested in this study, cultures at 160 cells/mm^2^ density were the only ones that permitted to obtain sparse neuronal network with individually identifiable neurons, statistically significantly impaired Ca^2+^ activity and clear dendritic atrophy in *Mecp2*^*−/y*^ neurons with respect to WT.

Finally, the method described in this study allows to use just one mouse brain (both hippocampi) to fill a 96 well plate. This finding is of general interest for *in vitro* research on cellular neuroscience, since many morphological studies are currently performed using high density cultures. A major strength of this method is that it could be also used to investigate multiple signaling pathways, since the 96 well plate format allows testing numerous experimental conditions.

In conclusion, we have successfully established an *in vitro* model of dendritic atrophy in *Mecp2*^*−/y*^ mouse hippocampal primary cultures that is suitable as a phenotypic drug screening assay because it shows significant reductions in total dendritic length, dendritic endpoints and soma size and responds with a rescue of these deficits after a three-day treatment with Mirtazapine and with a trophic response to BDNF. Being based on free and open-source software and consenting a high degree of automation, the method described here represents an accessible, powerful and quantitative tool for investigating virtually every neurodevelopmental disease with dendritic atrophy.

## Methods

### Mice strain and genotyping

In conformity to the Italian legislation D.Lgs. 116/92, animal use was approved by the Italian Ministry of Health (Authorization n. 71/2018-PR) and all experiments were performed in accordance with relevant guidelines and regulations. Animals were kept in ventilated cages under 12/12 h light/dark cycle with food and water *ad libitum*. Wild-type (WT) C57BL/6 male mice (Charles River Laboratories, Calco, LC, Italy) were crossed with C57BL/6 female mice heterozygous for the deletion of exons 3 and 4 in *Mecp2* gene^41^ (*Mecp2*−*/*+, B6.129P2(C)-Mecp2tm1.1Bird/J, stock: 003890, Jackson Laboratories, Bar Harbor, Maine) to obtain Wild-Type (*Mecp2*+*/y*, WT) and Knock-Out (*Mecp2*^*−/y*^, KO) male mice.

Mice genotype was determined using DNA extracted from tails using KAPA Express Extract Buffer and KAPA Express Extract Enzyme (KAPA Biosystems, Cape Town, South Africa). Polymerase Chain Reaction (PCR) was performed using KAPA2G Fast DNA polymerase with the following primers: 5′-AAATTGGGTTACACCGCTGA-3′ (Common Forward 9875, Jackson Laboratory), 5′-CTGTATCCTTGGGTCAAGCTG-3′ (Wild Type Reverse oIMR7172, Jackson Laboratory), 5′- CCACCTAGCCTGCCTGTACT-3′ (Mutant Reverse 9877, Jackson Laboratory). PCR reaction was performed in a final volume of 25 *µ*l set as follows: initial denaturation at 95 °C for 3 minutes then, 95 °C for 20 seconds, 58 °C for 20 seconds, 72 °C for 20 seconds (35 cycles) and final elongation at 72 °C for 2 minutes.

### Culture of hippocampal primary neurons

Hippocampal neuronal cultures were prepared from postnatal day 0 or 1 (P0–P1) Wild-Type (*Mecp2*^+*/y*^, WT) and Knock-Out (*Mecp2*^*−/y*^, KO) male mice as previously described^14^. Briefly, mice were decapitated and both hippocampi extracted and collected in cold Hank’s balanced salt solution HBSS (NaHCO3 4.2 mM, Hank’s salt powder 0.952%, HEPES 12 mM, 4-(2-hydroxyethyl)-1-piperazineethane-sulphonic acid, Sigma), then digested with 0.25% Trypsine (Euroclone) at 37 °C for 8 minutes. Enzymatic digestion was blocked with Dulbecco’s Modified Eagle Medium high glucose (DMEM, Euroclone), supplemented with 10% Fetal Bovine Serum (FBS) and penicillin-streptomycin (Euroclone), then the tissue was centrifuged at 800 rpm for 5 minutes at room temperature. The tissue was resuspended with DMEM + 10% FBS and mechanically triturated. Cells were counted with the dye exclusion method using Trypan Blue (Sigma) in the Burker chamber (Eppendorf), obtaining 600 000–800 000 cells from each mouse. Cells were plated either on 12 mm cover glasses (Sacco) or directly on polystyrene in 24-, 96- and 96 half surface-multiwell plates (Sarstedt), coated with 0.1% poly-L-Ornithine (Sigma) with/without 2% Matrigel (BD science). We seeded cells at different cell densities: 640 cells/mm^2^, 320 cells/mm^2^, 160 cells/mm^2^, 80 cells/mm^2^ and 40 cells/mm^2^. Cultures were grown at 37 °C and 5% CO_2_ in Neurobasal (Invitrogen) supplemented with 2% B-27 (Invitrogen), 1 mM L-glutamine and 1% penicillin-streptomycin. Cell medium was changed at DIV 3 including Cytosine *β*-D-arabinofuranoside (Ara-C, Sigma) at the final concentration of 2.5 *µ*M to inhibit proliferation of non-neuronal cells. Cells were maintained in culture until DIV 12–13. For the experiment shown in Supplementary Fig. 1, using 24-well plates, cells were infected at DIV 3 with an Adeno-Associated Virus (AAV9) expressing GFP under the synapsin promoter at the concentration of 5 × 10^13^ U/mL.

### Treatments

Drugs were included in culture medium at DIV 9 for 3 days. BDNF (Alomone Labs) was used at the final concentration of 25, 50 ng/ml, in Phosphate-buffered saline solution (PBS). Mirtazapine (Abcam Biochemicals; Cat. N. ab120068) was tested at the concentration of 1, 5 or 10 µM (in DMSO 0.1%).

### Neuronal morphology analysis

Soma area, TDL and number of endpoints were measured using NeuriteQuant, an open source plugin for ImageJ^42^. The analysis is independent from concentration of 25, 50 ng/ml, in Phosphate-buffered saline solution (PBS) which was added to the culture medium (50 µl of 4xBDNF in 150 µl of Neurobasal + B27 medium = 200 µl total volume). Instead the control samples were incubated with 50 µl PBS (the vehicle for BDNF) in 150 µl of Neurobasal + B27 medium ( = 200 µl total volume). Mirtazapine (abcam Biochemicals; Cat. N. ab120068) was tested at the concentration of 1, 5 or 10 *µ*M (in DMSO 0.1%).

### Immunofluorescence

Hippocampal primary cultures were fixed at DIV 12 using 4% Paraformaldehyde (PFA, Sigma) in PBS for 15 minutes at room temperature, then washed with PBS and permeabilized using PBS-Triton 0,1% for 25 minutes. To block unspecific binding sites, blocking solution was prepared with PBS-Triton 0,1% and 2% Bovine Serum Albumin (BSA, Sigma). Primary antibodies were diluted in blocking solution (see Table 1 for dilutions) and incubated for 1 hour at room temperature in dark humified chamber. Cells were washed twice with PBS-Triton 0,1% and incubated with secondary antibodies diluted 1:500 in blocking solution: anti-rabbit IgG Alexa Fluor568 (Invitrogen), anti-mouse IgG Alexa Fluor647 (Invitrogen) and anti- mouse IgG Alexa Fluor488 (Invitrogen). Cells were washed twice with PBS-Triton 0,1% and incubated with Hoechst 33342 (10 mg/ml, Sigma) at the dilution 1:1000 (final concentration 10 *µ*g/ml) in PBS for 5 minutes. Glass coverslips were washed with MilliQ water and Mowiol (Sigma) was added to preserve fluorescence, then were left at room temperature overnight and imaged the day after, while polystyrene plates were left in PBS and imaged within a few hours. In Supplementary Fig. 1 is shown an example of the differences in average Total Dendritic lenght (TDL) measured using GFP-staining with AAV9 and different anti-MAP2 antibodies at the cellular concentration of 160 cells/mm2 at DIV 12.

**Table 1:** Primary antibodies used.

Antibody	Species	Dilution	Company	Code
anti-MAP2 isotypes 2 A + 2B + 2C	Rabbit pAb	1:500	SantaCruz	Dismissed
anti-MAP2 isotypes 2A + 2B	Mouse mAb	1:500	Sigma	M1406
anti-MAP2 isotypes 2A + 2B + 2C + 2D	Rabbit pAb	1:500	Genetex	GTX50810
anti-NeuN	Mouse mAb	1:1000	Millipore	MAB377

Images were acquired using Nikon Eclipse Ti-E epifluorescence live imaging microscope equipped with a motorized stage and a Nikon DS-Qi2 camera (CMOS sensor, 16.25 megapixel, 14 bit gray levels). Acquisitions were performed using the software Nis-Elements 4.60 with the module “JOBS” for automated imaging. For the analysis of untreated WT and *Mecp2*^*−/y*^ neurons (Figs. 1–4), four images were acquired with 20x in the central region of each well and combined into a single large image by the function “Stitching” of the Nis-Elements software. For the analysis of the cultures treated with BDNF or Mirtazapine (Figs. 6, 7), seven random fields per well were acquired with 10x objective and analyzed individually. The number of neurons and the total number of cells seeded was determined counting NeuN positive cells and Hoechst positive cell respectively, with the “Object-analyzer” plugin for Nis-Elements 4.60.

### Neuronal morphology analysis

Soma area, TDL and number of endpoints were measured using NeuriteQuant, an open source plugin for ImageJ. The analysis is independent from signal intensity, so it can trace neurites which show both a strong or a weak signal intensity. Neverthless, a signal-to-noise ratio higher than 3 is preferred and somehow required^42^. To perform the analysis, there are four different parameters to be set and a correct tuning of these parameters is required in order to achieve a reliable analysis. We set these parameters as follows:

1. *Neurite detection width*: 12.
2. *Neurite detection threshold*: 8.
3. *Neurite cleanup threshold*: 170.
4. *Neuronal cell body detection*: 300.

### Cell cluster analysis

To characterize the differences in the distribution of cells within a well at the different cell densities and in the different well plates, we acquired images of the full well surface in the Hoechst channel (nuclear staining) using 4X objective (2, 20 *µ*m/px resolution), both for WT and *Mecp2*^*−/y*^ cells. These images were used to calculate the *Cell Cluster Index*, which represents a quantitative evaluation of the degree of clustering of the cells, based on the “nucleus-nucleus distance Index”. We used the ClusterIndex Jython script designed by Dr. Kota Miura (available here: ClusterIndex-guithub), that we have customized to analyze a region of interest (ROI) excluding from the analysis the borders of the wells.

### Calcium imaging

Cells for calcium imaging were plated at 640 cell/mm^2^, 320 cell/mm^2^, 160 cell/mm^2^ and 80 cell/mm^2^. Experiments were performed at DIV 12–13, on both WT and *Mecp2*^−/y^ cells. The culture medium was removed, and cells were loaded with the calcium indicator acetoxymethyl ester Oregon green 488 BAPTA-1 (Thermofischer) diluted in KREBS solution (NaCl 150 mM, KCl 4 mM, CaCl2 2 mM, MgCl2 1 mM, HEPES 10 mM, D-Glucose 10 mM, pH 7.4) 1% BSA to reach the final concentration of 10 µM. Once loaded, cells were kept for 2–3 hours at 37 °C and then the loading solution was replaced with fresh KREBS solution. Images were acquired using Nikon Eclipse Ti-E-epifluorescence live imaging microscope using 20 X objective. The software acquisition used was Nis-Element 4.60, with an exposure time of 250 msec and a frame-rate of 3 images/sec (i.e. every 330 msec). The acquisition protocol provides for each well 3 acquisitions lasting 3:30 minutes, interleaved by 10:30 minutes of no acquisition. We performed a further 7 minutes-long acquisition, in which cell were stimulated with KCl (final concentration 50 mM) followed by a pause of 7 minutes and then 3:30 minutes acquisition. To maintain the physiological conditions through the entire acquisition process, the plates were mounted in stage top incubator maintaining the temperature at 37 °C and receiving 5% CO2 balanced with air by a flow meter (Okolab, Pozzuoli-Napoli, Italy).

### Statistical analysis

All statistical data analysis and data representation were performed using the Prism 6 software (Graphpad). Statistical significance for comparisons between different groups was established using either a Student t-test when comparing 2 conditions/group, One-Way ANOVA for multiple comparisons to compare more than 2 groups or Two-way ANOVA to compare the effects of 2 independent variables (e.g. cell density and genotype). In the case of data not passing the normality test (Shapiro-Wilk) the statistical difference was calculated using Mann-Whitney Rank Sum Test to compare 2 groups, or Kruskall Wallis test when comparing more than 2 groups. Outlier’s detection was performed using Grubb’s test with Graphpad software. For Ca^2+^ imaging, the Interevent interval statistical analysis was performed using the Kolmogorv-Smirnov test (Graph Pad Prism 7.00).

## Supplementary information

.Supplementary Information.

## Data Availability

The datasets generated for this study are included in the manuscript.
