# Supplementary material for: In vitro modeling of dendritic atrophy in Rett syndrome: determinants for phenotypic drug screening in neurodevelopmental disorders
Source: Sci Rep. 2020 Feb 12;10:2491. doi: 10.1038/s41598-020-59268-w (PMC7016139; doi:10.1038/s41598-020-59268-w)
Supplement: Supplementary file 1 — .Supplementary Information. [file 41598_2020_59268_MOESM1_ESM.pdf]

SUPPLEMENTARY MATERIAL:

"In vitro modeling of dendritic atrophy in Rett syndrome: determinants for phenotypic drug screening in neurodevelopmental disorders"

NERLI Elisa, ROGGERO Ottavia, BAJ Gabriele and TONGIORGI Enrico

BRAIN Center for Neuroscience, Department of Life Sciences, University of Trieste, 34127 Trieste-Italy

This file contains:

- Supplementary Figure 1.
- Supplementary Figure 2.
- Supplementary Figure 3.
- Supplementary Table 1.

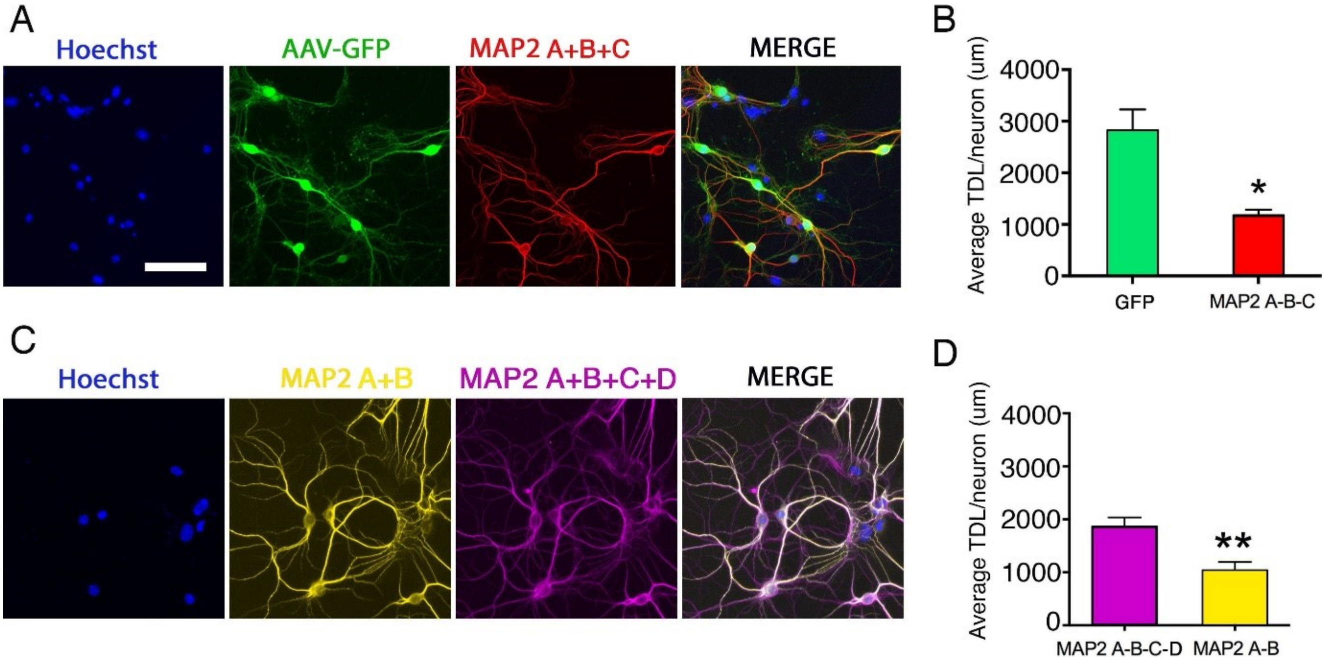

Supplementary Figure 1. Comparison between different stainings. Panel A: AAV9-GFP and rabbit polyclonal anti-MAP2 1:500 isotypes A+B+C (SantaCruz, dismissed) stainings and comparison between the TDL measures (panel B). Panel C, mouse anti-MAP2 1:500 isotypes A+B (Sigma M1406) and rabbit anti-MAP2 1:500 isotypes A+B+C+D (Genetex, GTX50810), and the comparison between the TDL measures (panel D). Scale bar = 100 μm.

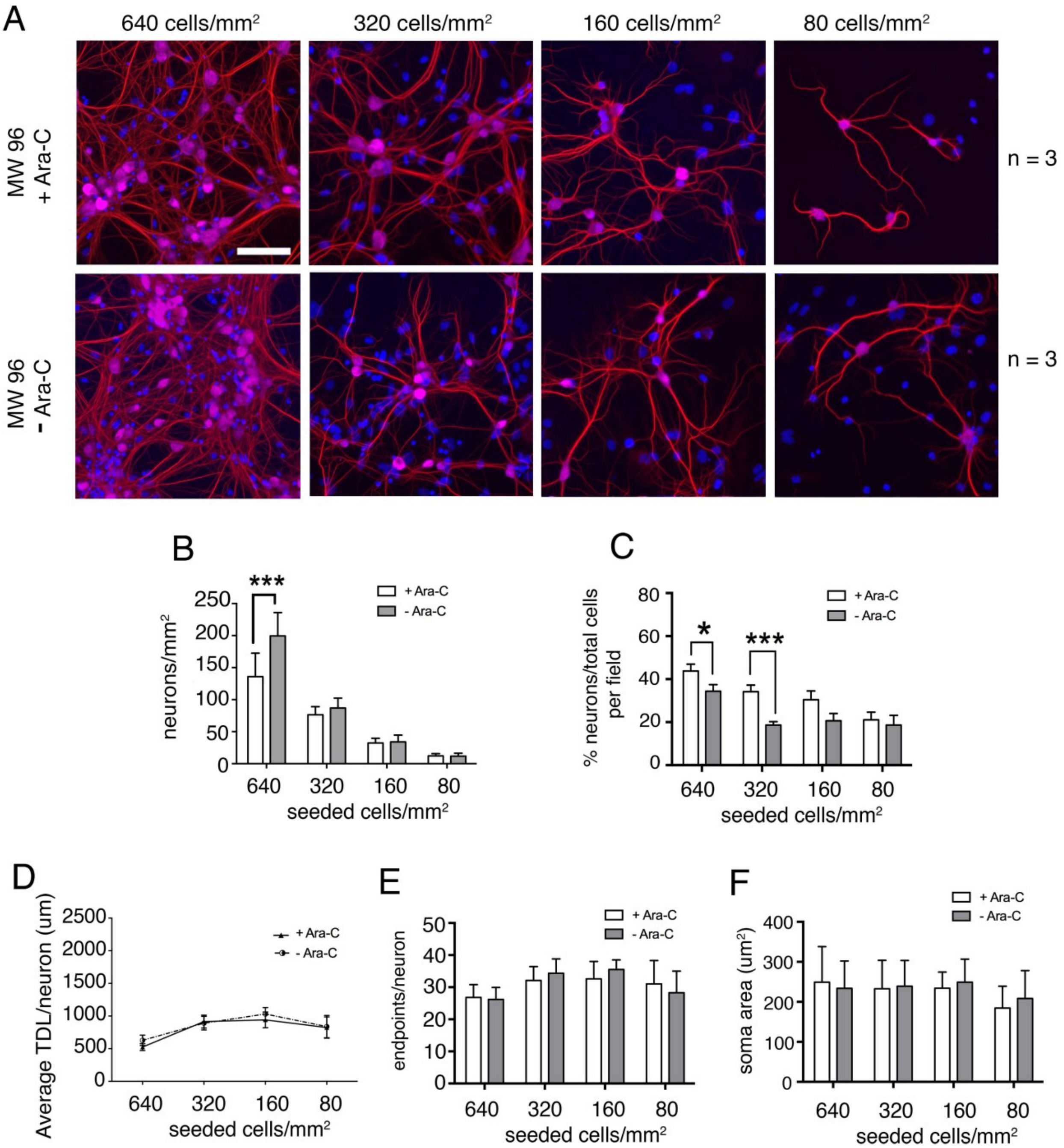

Supplementary Figure 2. Morphological parameters in presence or absence of Ara-C. (A) DIV12 mouse hippocampal neurons immunostained for cytoskeleton (MAP2 red) and nuclei (Hoechst blue) at different seeding cell densities with Ara-C 2,5 μM (top line) or without Ara-C (bottom line) on 96 well plates (Scale bar = 100 μm). (B) Quantitative data on the number of neurons per mm^2 counted at the different seeding cell densities. Data are expressed as mean ±SEM, n=3 mice per condition. (C) Number of neurons (%) normalized on the number of counted viable nuclei (total cells), (D) average TDL per neuron (μm), (E) average number of endpoints per neuron and (F) soma area per neuron (μm^2). Number of neurons measured ranged from 1000 for the highest seeded cell density, down to 200 neurons for the lowest seeded cell density. t-test to compare the 2 different conditions (+ Ara-C or − Ara-C) at each cellular concentration: ns P>0.05, * P≤0.05, ** P≤0.01, *** P≤0.001.

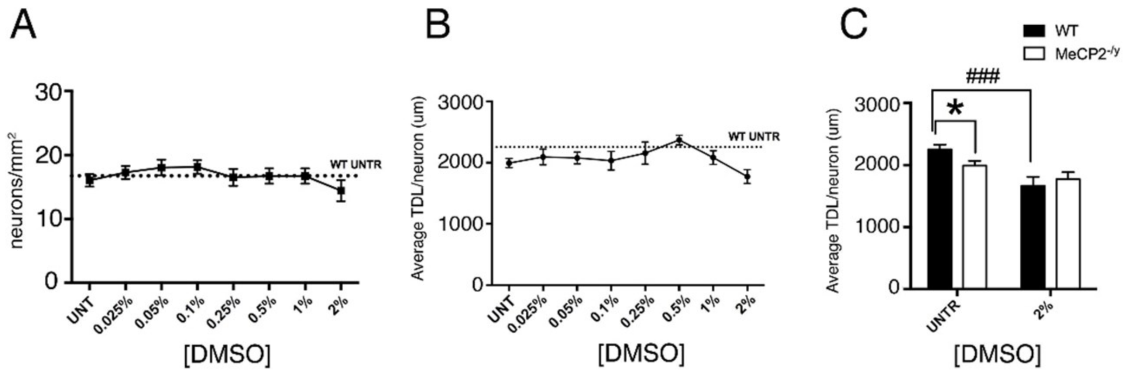

Supplementary figure 3. DMSO effect on neuron viability and morphology. Effect of increasing concentration of DMSO on neuron number (panel A) and average TDL (panel B) on *MeCP2*$^{-/y}$ neurons seeded at 160 cells/mm^2. Dotted line is referred to WT untreated, which means WT neurons maintained in Neurobasal + B27 for the whole experiment. In figure C, a detail of average TDL at 2% DMSO with respect to WT untreated for WT and *MeCP2*$^{-/y}$ neurons (i.e. maintained in Neurobasal + B27). One-way ANOVA to compare the effect of different concentration of DMSO in figure A and B: ### P≤0.001. t-test to compare WT and *MeCP2*$^{-/y}$ neurons in figure C: * P≤0.05. For each condition, 20 wells per WT and 12 wells per *MeCP2*$^{-/y}$ neurons were analyzed (1 field per well, 1800 and 1000 neurons, respectively). n=9 wells for UNTR condition (800 neurons), n=3 wells for the other conditions (270 neurons).

Supplementary Table 1

Boltzmann sigmoidal Best-fit values

	WT 640	WT 320	WT 160	WT 80
**Slope**	1.85	5.789	10.88	8.222
**Std. Error**	0.1193	0.5477	2.221	2.129

Boltzmann sigmoidal Best-fit values

	MeCP2 KO 640	MeCP2 KO 320	MeCP2 KO160	MeCP2 KO 80
**Slope**	7.903	10.94	3.278	0.09788
**Std. Error**	1.198	3.204	0.4467	0.1038

Supplementary Table 1. Best-fit values of spontaneous calcium spike activity, shown in Figure 5D.
